# Fœtal Psychology

**Published:** 1886-05-20

**Authors:** Thos. S. Sozinskey

**Affiliations:** Philadelphia


### FŒTAL PSYCHOLOGY.

BY THOS. S. SOZINSKEY, M.D., PH. D., OF PHILADELPHIA.

"We may fairly assume from analogy that a long time before it is born a child will have become acquainted both with pain and pleasure." Thus it is said on page 7 of an excellent little work by M. Bernard Perez, a translation of which, with a flattering introduction by the distinguished English psychologist, James Sully, has just been published, entitled "The First Three Years of Childhood." The question is not only curious, but highly interesting, especially to physicians. If an unborn child may experience both pleasurable and painful sensations, the fact should be more widely known than it seems to be. I am of the belief that medical graduates are oblivious of it. I am pretty sure I never had it brought to my attention in a lecture on obstetrics.

M. Perez is by no means a visionary theorist.; on the contrary, he is a patient, sharp observer, and in full sympathy with the modern scientific school of philosophy. Mr. Sully remarks of him that he "combines considerable physiological and psychological knowledge with a practical interest in education." What he may have to say is certainly entitled to respectful attention. To be sure, he is not a physician; but it may not be doubted that a practical educationist may be quite familiar with the facts of psychology. Still, there are some statements in M. Perez's book which he would hardly have made had he been an accoucheur. Thus, says he, "there is no doubt that the moment when the infant first enters into relation with external realities is a very painful one."

Of very ancient date, indeed, is the belief that unborn babies may experience pleasure and pain. There is a well-known passage of Scripture (St. Luke i. 44) illustrative of it. It is upheld by many theologians, as well as "old ladies," and others at the present day.

Notwithstanding the positive statements of M. Perez and others, I think nearly all physicians hold that intra-uterine life is a blank mentally. Before birth, a child can experience no sensation whatever, either pleasurable or painful. There is no fœtal psychology. So the doctor is apt to think: but can he present sufficient reasons for his belief?

The question is really an open one, I believe. That an unborn child is alive is certain. It is equally certain that without nerve-centres in full play there could be no pulsation of the heart or any other action of the child's economy. No doubt all pre-natal activities may be very reasonably classed as reflex; but there is no very evident reason why the cerebral activity on which consciousness depends should not at times exist, at least toward the end of the period of gestation.

Is there no stimulus to excite consciousness in the fœtal brain? There is sufficient ground for believing that if the brain were without external connections there could be no mental activity within it; without the special senses there could be no mind. In the fœtus the senses of sight, smell, taste, and perhaps hearing, are entirely dormant, but what of a touch, or rather general sensibility? May not a marked disturbance of function or impression made on the skin of a fœtus be productive of consciousness? I believe all the necessary conditions of nerves and brain are present in the later months of fœtal life. Is it reasonable then, to declare consciousness in the fœtus, at any time, to be impossible?

But from what we know of the degree of sensibility of new-born babies, it is highly improbable that vivid consciousness is possible in the fœtus, even at term. In general, there is but little chance during intra-uterine life for decided local impressions to be made; and most of such, it may be observed, point to pain. Jars and other rude impressions are only occasionally experienced, and it is only from these likely that the production of any degree of consciousness is at all probable. During labor the pressure experienced by the head is usually sufficient to effectually preclude any possibility of consciousness being aroused; and hence birth cannot, as a rule, be a painful process.

Thus much for the present on the subject of fœtal psychology. It is of enough importance, I believe, to receive some attention from medical men.—*Medical and Surgical Reporter.*

